# Linear discriminant analysis reveals differences in root architecture in wheat seedlings related to nitrogen uptake efficiency

**DOI:** 10.1093/jxb/erx300

**Published:** 2017-09-27

**Authors:** Kim Kenobi, Jonathan A Atkinson, Darren M Wells, Oorbessy Gaju, Jayalath G De Silva, M John Foulkes, Ian L Dryden, Andrew T A Wood, Malcolm J Bennett

**Affiliations:** 1 Department of Mathematics, Aberystwyth University, Penglais, Aberystwyth, Ceredigion; 2 Centre for Plant Integrative Biology, School of Biosciences, Sutton Bonington Campus, University of Nottingham, UK; 3 Division of Plant and Crop Sciences, Sutton Bonington Campus, University of Nottingham, UK; 4 School of Mathematical Sciences, University of Nottingham, University Park, UK

**Keywords:** Linear discriminant analysis, Mahalanobis distance, nitrogen uptake efficiency, plant phenotyping, root system architecture, Watkins lines, *Triticum aestivum*, wheat root biology

## Abstract

Root architecture impacts water and nutrient uptake efficiency. Identifying exactly which root architectural properties influence these agronomic traits can prove challenging. In this paper, approximately 300 wheat (*Triticum aestivum*) plants were divided into four groups using two binary classifications, high versus low nitrogen uptake efficiency (NUpE), and high versus low nitrate in the growth medium. The root system architecture for each wheat plant was captured using 16 quantitative variables. The multivariate analysis tool, linear discriminant analysis, was used to construct composite variables, each a linear combination of the original variables, such that the score of the plants on the new variables showed the maximum between-group variability. The results show that the distribution of root-system architecture traits differs between low- and high-NUpE plants and, less strongly, between low-NUpE plants grown on low versus high nitrate media.

## Teaser

Feature comparison of wheat seedlings obtained from high-throughput phenotyping using linear discriminant analysis shows that nitrogen uptake efficiency and nitrate availability affect root system architecture.

## Introduction

Plant phenotyping is becoming an important aspect of plant biology, as the global community of plant and crop scientists responds to the challenge of feeding a population that is predicted to be 9 billion people by the year 2050. Plant roots present a particular challenge in terms of the phenotyping effort, since it is difficult to make measurements of the below-ground parts of plants. Plant roots serve a number of functions, including water and nutrient uptake, anchorage, photoassimilate storage, phytohormone synthesis, and clonal propagation. Root system architecture (RSA) is a highly plastic trait that enables plants to respond to changes in bioavailability of water and nutrients in the soil in order to optimize nutrient uptake efficiency. Two main root system morphologies are recurrent in angiosperms, namely the allorhizic system typically found in eudicot species and the secondary homorhizic system of monocot species. Allorhizic root systems are dominated by the primary root, which produces lateral roots that can form higher-order lateral roots. The secondary homorhizic root system is characterized by the development of many adventitious roots in parallel to the primary root (Osmont *et al.*, 2007; Atkinson *et al.*, 2014).

The importance of RSA in modulating a plant’s capacity to absorb nutrients efficiently is demonstrated by a wealth of both experimental and simulation-based evidence that demonstrates how RSA changes in response to nutrient treatment regimes. Experimental examples include changes in RSA in response to the distribution of phosphate (Williamson *et al.*, 2001), nitrate (Linkohr *et al.*, 2002), and water (Tsutsumi *et al.*, 2003). In a simulation model described in Dunbabin *et al.* (2004), a sparsely branched (herringbone) architecture was found to have a higher nitrate uptake efficiency than a highly branched (dichotomous) architecture when nutrient supply varied spatially and temporally.

Ideotypes have been proposed for RSA to optimize water and N acquisition. For example, Lynch (2013) describes an ideotype for maize that includes a large-diameter primary root with few but long laterals, tolerance of cold soil temperatures, and many seminal roots with shallow growth angles and long root hairs. In Foulkes *et al.* (2009), the authors conclude that increased root length density at depth may be associated with high capacities for uptake and assimilation of N in wheat plants.

Due to technical constraints, high-throughput root phenotyping is usually based on seedling screens using artificial media, the results from which may not relate to performance of mature plants in soil. However, several studies have shown significant correlations between seedling and field traits (Watt *et al.*, 2013; Canè *et al.*, 2014; Li *et al.*, 2015). Bai *et al.* (2013) used a paper-roll culture system to investigate root morphology in a mapping population of wheat and measured a suite of quantitative root traits on seedlings. Some of these, e.g. total root length and length of seminal laterals, are also used in the analysis presented here. Others, including surface area of seminal laterals and total root volume, were not available in this current analysis, since the data were two-dimensional (2-D) images. The root traits in Bai *et al.* (2013) were considered individually, and correlations with phenotypic traits including plant height and root to shoot ratio were calculated. The authors noted that plant height and root proliferation were not simply related.

A software tool called RootNav (Pound *et al.*, 2013) has recently been developed to help plant biologists with the quantification of 2-D seedling root systems. It allows for the collection of a wide range of measurements on root systems in a semi-automated way. The traits that can currently be measured include the number and lengths of both seminal and lateral roots, the emergence and tip angles of the roots, the area of the convex hull, and the maximum width and depth of a root system. RootNav is written in a modular way in the programming language C# and it is therefore straightforward to introduce modules for other traits as required. In addition to the quantitative traits mentioned above, RootNav can also output data about the spline curves that are fitted to the seminal and lateral roots. These are output as sets of co-ordinates of a large number of points along each root.

The problem of registration of biological images is a common one. Typical contexts in human medicine include the matching of ultrasound breast images (Neemuchwala *et al.*, 2001), registration of 3-D cerebral vessels (Bullitt *et al.*, 1999), registering CT scans of the lungs (Li *et al.*, 2003), and registration of retinal images based on reconstructed vascular trees (Fang and Tang, 2006). In plant biology, there is also considerable interest in this topic, for example the automatic registration of optical and infrared images of plant canopies (Yang *et al.*, 2009) or the use of MRI images to reconstruct plant root systems (Schulz *et al.*, 2013).

Of the above image-analysis contexts, many include working with branched structures (mathematical trees). The challenges in working with such data include optimal alignment of images and finding useful distance (or similarity) measures between the trees. Here, a novel distance measure between two root systems is constructed and used to perform multidimensional scaling (MDS) on the data set. The MDS co-ordinates in five dimensions along with 11 quantitative variables are used. Linear discriminant analysis (LDA) is used in combination with a subset selection package in R (www.r-project.org) to identify a subset of the variables that best discriminates between the four nitrogen uptake efficiency (NUpE)/nitrate treatment combinations of wheat lines (low versus high NUpE and low versus high nitrate in the medium).

In this paper, a statistical analysis of a data set of 2-D images of the seedling root system of wheat (*Triticum aestivum*) plants grown in growth pouches in controlled environment conditions is presented. The analysis makes use of the geometric data in the spline co-ordinates as well as the set of quantitative traits obtained from RootNav. The data consist of measurements on the seedling roots for nine different wheat lines. The analysis reveals highly significant and robust differences in the structure of the data sets corresponding to field measurements of low and high NUpE, indicating that the root system architecture of the wheat seedlings is different for low- versus high-NUpE lines. This approach allows combinations of seedling root traits not readily observable by eye to be related to the field performance of mature plants.

The aim of this paper is to demonstrate that in multivariate data sets obtained from high-throughput plant phenotyping experiments, there may be structural patterns in the data that are not easily discernible by eye. In this study, the use of linear discriminant analysis revealed clear differences in the distributions of root system architecture traits between wheat plants classified as low or high NUpE on the basis of field trials. In addition, within the low-NUpE wheat plants, linear discriminant analysis revealed that the distribution of root system architecture traits differed between plants grown on low-nitrate versus high-nitrate media.

## Materials and methods

### Plant materials

The nine wheat accessions, W1190145, W1190149, W1190199, W1190325, W1190483, W1190637, W1190685, W1190700, and W1190705, were selected from the Watkins collection (see Miller *et al.*, 2001, for details). In brief, the Watkins lines are selections of landrace wheats collected from 32 countries around the world in the late 1930s by E.A. Watkins. The collection offers a unique snapshot of genetic diversity and geographic distribution prior to modern plant breeding and the green revolution.

### Field trials

The nitrogen uptake efficiency data are based on experiments at the University of Nottingham in 2010–2012 (for details see Gaju *et al.*, 2016). Plants were sown using a split-plot design in which N fertilizer treatment was randomized on main plots and genotype was randomized on the sub-plots in three replicates. The concentration of N in the straw and the grain was measured in each sub-plot in each experiment using the Dumas method on hand-harvested samples. The nitrogen uptake efficiency (NUpE) was calculated by dividing the above-ground N at harvest (kg N ha^−1^) by the amount of N available to the crop from the soil N and fertilizer (kg N ha^−1^). The NUpE of the nine lines on the low-nitrogen treatment plots in the growing season 2010–11 was used as the basis for initial characterization of the lines as low or high NUpE. Their performance was comparable in the season 2011–12, in the sense that lines initially characterized as being low NUpE in the first season showed lower nitrogen uptake under low-nitrogen treatment in 2011–12 than the lines characterized as being high NUpE. For details of the data used for the low/high NUpE characterisation see Supplementary Fig. S1 at *JXB* online.

### Root system phenotyping

**Fig. 1.**
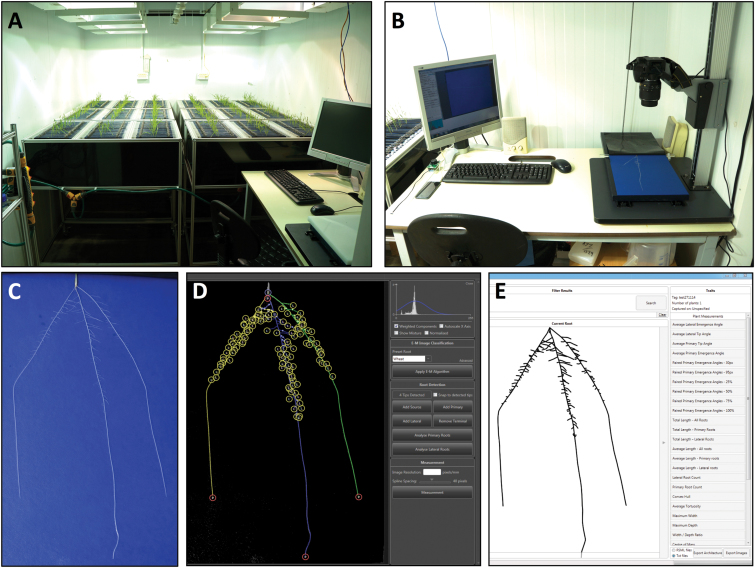
Seedling root phenotyping pipeline. (A) Growth assembly. (B) Image acquisition. (C) Example root image. (D) Root system extraction and quantification using RootNav software. (E) Reconstruction of root system *in silico* and trait quantification. Figure adapted from Atkinson *et al.* (2015).

The set-up of the phenotyping platform used is shown in Fig. 1. Seeds were surface-sterilized by incubation in 70% (v/v) ethanol for 30 s, followed by transfer to 5% (v/v) sodium hypochlorite solution for 10 min, and finally rinsed three times with sterile water. Sterilized seeds were placed onto moistened germination paper, crease-side down, and incubated at 4 °C for 5 d to synchronize germination. Following this cold treatment, seeds were transferred to a light-impermeable box for 48 h to complete germination. This box was placed inside the controlled environment room (12 h photoperiod, 20 °C day, 15 °C night, at a light intensity of 400 µmol m^–2^ s^–1^ PAR) where subsequent phenotyping was conducted. Uniformly germinated seeds with roots approximately 5 mm in length were transferred to growth pouches. Each pouch consisted of a sheet of germination paper (24 × 30 cm; Anchor Paper Company, St. Paul, MN, USA), covered with a black polythene film of equal area (75 µm thick; Cransford Polythene LTD, Suffolk, UK). The germination paper and film were fixed to an acrylic rod (316 × 15 × 5 mm; Acrylic Online, Hull, UK) using two 18-mm foldback clips. A QR code label affixed to the rod allowed identification of each seedling. A single seedling was placed in each pouch, centred 2 cm from the top edge and held in place by the adhesion of the polythene sheet to the wet germination paper. Growth pouches were fitted into four aluminium and polypropylene frame assemblies in the controlled environment chamber. Each assembly consisted of an aluminium profile frame (104 × 62 × 102 cm; KJN Ltd, Leicester, UK) supporting toothed acrylic holders to suspend each pouch in a set position. Black polypropylene side panels (101 × 31cm × 0.3 cm and 63 × 31 × 0.3 cm; Cut Plastic Sheeting, Devon, UK) maintained the pouches in darkness. The base of each frame held a black polypropylene tray (99 × 61 × 10 cm; Baker Environmental Lining Services LTD, Essex, UK) containing 18 l of ¼ Hoagland’s solution (Hoagland and Arnon, 1950) with HEDTA as the iron chelator (Piñeros *et al.*, 2005). The nutrient solution level in each tray was automatically maintained by a float-valve system and a header tank containing reverse osmosis water. Each frame assembly consisted of three rows of 30 pouches, allowing 90 plants per frame. Pouches were suspended so that the bottom 3 cm of the pouch was submerged in the nutrient solution.

After 9 d (2-leaf stage), individual pouches were transferred to a copy stand (model number SGCS-920, Speed Graphic, Hampshire, UK) for imaging with a Nikon D600 DSLR camera. The copy stand was modified with two draw-slides (RS UK, Northants, UK), a Nylatron block (600 × 260 × 22 mm), and white acrylic sheets (330 × 290 × 9 mm and 290 × 290 × 9 mm; Cut Plastic Sheeting, Devon, UK) to form a template to ensure identical placement of each pouch. The polythene film covering each pouch was carefully peeled back leaving the roots fixed to the germination paper for imaging. The draw-slides then enable the template block to be repositioned, allowing shoots to be imaged.

### Image analysis

The resulting images were analysed using the software package RootNav (Pound *et al.*, 2013). Eleven quantitative traits from RootNav were used for this analysis: Total Length, Average Seminal Tip Angle, Average Seminal Emergence Angle, Average Length – Seminal Roots, Average Length – Lateral Roots, Lateral Root Count, Seminal Root Count, Convex Hull, Maximum Width, Maximum Depth, Width-Depth Ratio. and In addition, five geometric variables were used, which were generated using the smoothing splines that RootNav fits to seminal and lateral roots combined with a distance measure (see Supplementary Methods S1) and multidimensional scaling (Supplementary Methods S2). The data are available in Supplementary Table S1 (raw data) and Table S2 (data scaled so each variable has mean 0 and variance 1). The latter (scaled) data were used in this analysis.

## Results

### Root phenotypic analysis of high- and low-NUpE Watkins lines

**Fig. 2.**
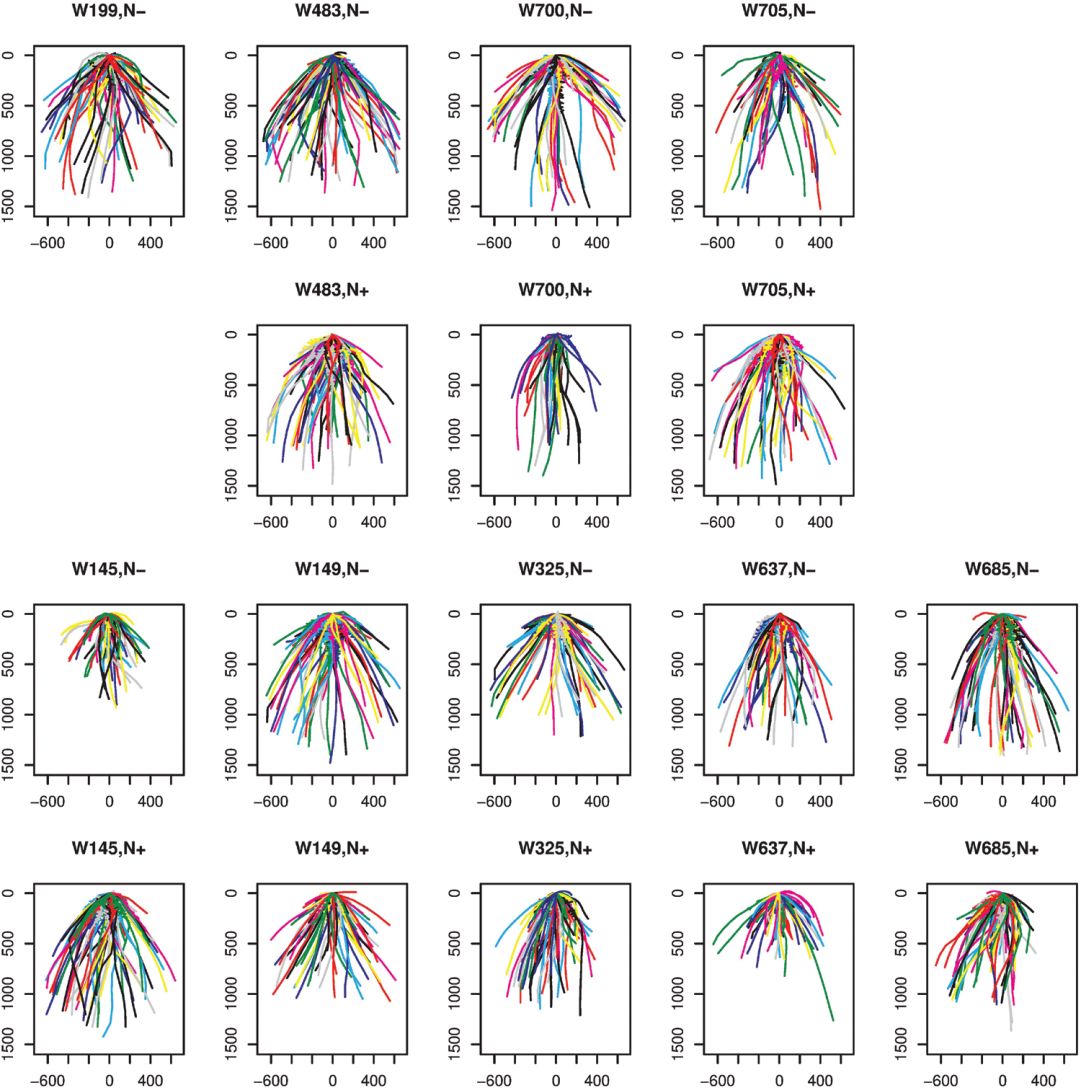
Overview of the root system architecture of the Watkins lines used in this analysis. Row 1: low-NUpE lines, low nitrate; Row 2: low-NUpE lines, high nitrate; Row 3: high-NUpE lines, low nitrate; Row 4: high-NUpE lines, high nitrate. In each plot, all of the root systems of plants in that combination of line and nitrate treatment are overlaid. Moving from Row 1 to Row 2 or from Row 3 to Row 4, the same lines under the different treatment conditions are in the same column. Data are not available for line W199 grown in a high-nitrate medium.

Field trials were used to establish the nitrogen uptake efficiency (NUpE) of the Watkins lines. From these trials, four low-NUpE lines and five high-NUpE lines were selected. For three of the low-NUpE lines and all of the high-NUpE lines, data under both low-and high-nitrogen growth conditions are available. The NUpE data for the nine lines across a 2-year Nottingham-based field study (Gaju *et al.*, 2016) are shown in Supplementary Fig. S1. In order to obtain an overview of the root system architecture of the approximately 300 wheat plants in the data set, the root systems of the plants for each line and each treatment condition are overlaid in Fig. 2. Between rows 1 and 2 and also between rows 3 and 4, the same wheat line occurs in each column, with low nitrate in rows 1 and 3 and high nitrate in rows 2 and 4. For some of the lines, Fig. 2 enables an obvious visual comparison. For example, comparing the two nitrate treatment conditions, for lines W700 and W325 the plants grown on a high-nitrate medium are narrower. In line W145 the low-nitrate plants are shallower, and for line W637 the high-nitrate plants are shallower. But, in general, it is difficult to discern clear differences in root system architecture from the images in Fig. 2.

### Revealing differences between lines and treatments

On each root, 16 quantitative variables were available: 11 measurements from RootNav and five geometric variables from the application of multidimensional scaling (see Supplementary Methods S1 and S2). Further, the roots were grouped, by line and nitrate treatment. Initially, principal component analysis (PCA) was applied to the data. This did not reveal any clear patterns (Supplementary Fig. S2).

Given two groups with the same set of measurements on each element of each group, the Mahalanobis distance can be used to calculate a distance between the two groups. The Mahalanobis distance is a multivariate generalization of the *t*-distances used in the familiar *t*-tests, and allows for the calculation of a distance between two samples that takes the covariance structure into consideration. For the mathematical details of the Mahalanobis distance see Supplementary Method S3).

**Fig. 3.**
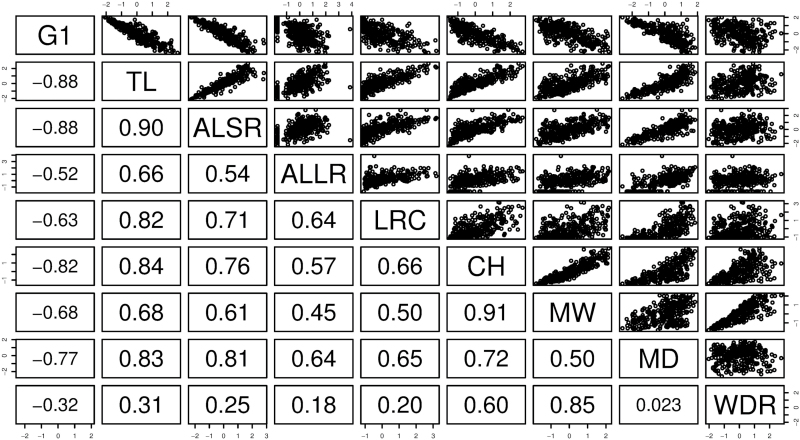
The pairwise correlations of all of the root variables for which the correlation with at least one other variable is of magnitude at least 0.5. The lower-left panels show the numerical values of the correlations (rounded to two decimal places). The upper-right panels show the scatter plots. All variables are scaled to have a mean of zero and a variance of 1. Abbreviations: G1, Geom1; TL, total length; ALSR, average length – seminal roots; ALLR, average length – lateral roots; LRC, lateral root count; CH, convex hull; MW, maximum width; MD, maximum depth; WDR, width–depth ratio.

The pairwise correlations between the variables are shown in Fig. 3 (only the variables for which the correlation with at least one other variable is of magnitude at least 0.5 are included). Geom1 (labelled G1 on the figure), the first of the shape variables obtained from the multidimensional scaling, is inversely correlated with various measures of ‘size’ (total length, average length of seminal roots, lateral root count, convex hull area, maximum width, and maximum depth.) There are a number of strong positive correlations among the other variables, such as convex hull and maximum width, and total length and lateral root count. These positive correlations are not surprising, but it is important to bear in mind that the traits generated by RootNav are not linearly independent.

**Fig. 4.**
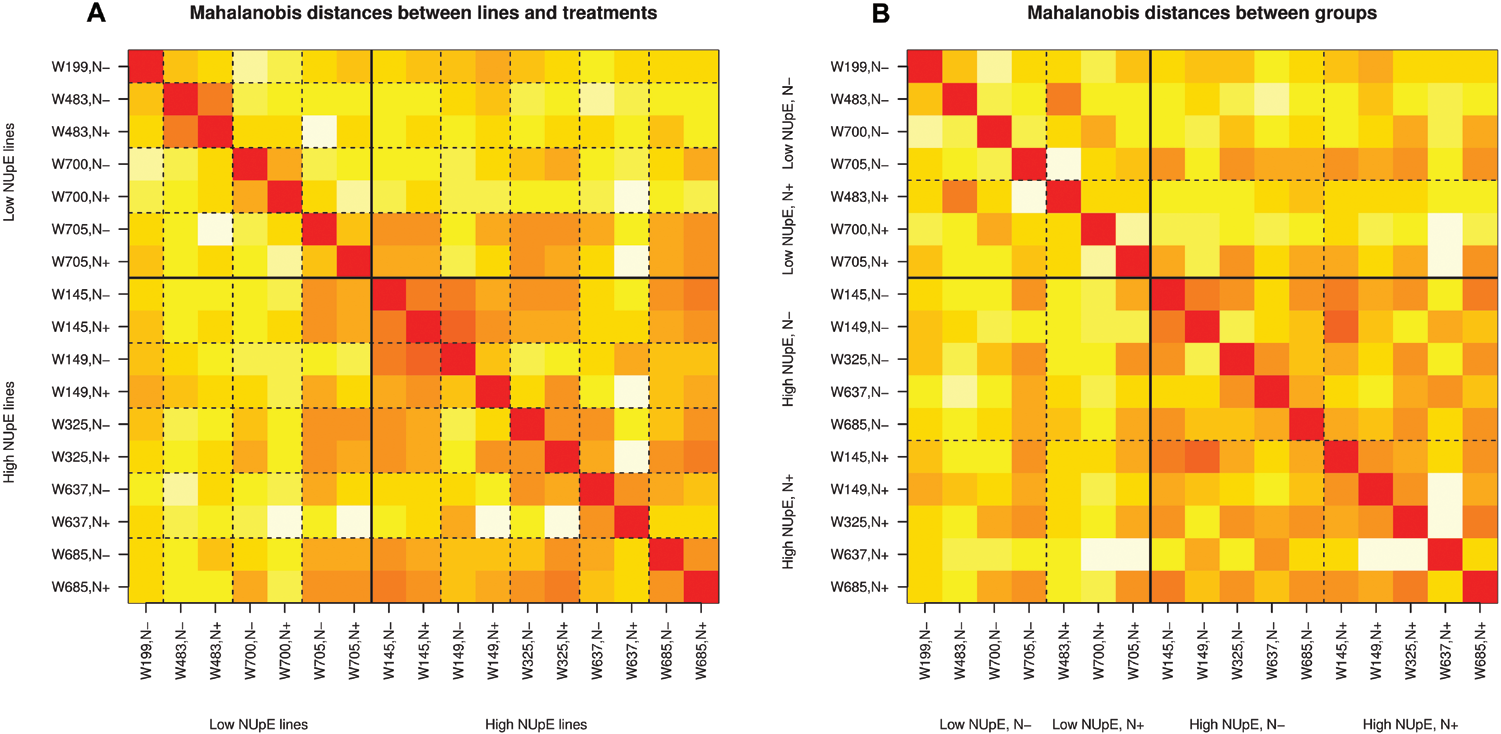
The Mahalanobis distances between samples corresponding to different lines and treatments. In (A) the columns or rows for the same line under low (N–) and high (N+) nitrate growth conditions are next to each other. In (B) the lines are grouped by nitrate treatment. The darker the square the smaller the distances between groups. Different patterns can be seen depending on how the lines are grouped.

Heat maps of the Mahalanobis distances between the lines and treatments in the data set are shown in Fig. 4. The two plots in the figure show the same data arranged in two different ways to highlight different features. The darker the colour, the smaller the distance between the samples. Within each wheat line, the low-nitrate sample is close to the high-nitrate sample as measured by the Mahalanobis distance (i.e. within the 2 × 2 squares marked out by the dashed lines in the heat map of Fig. 4A the off-diagonal elements are quite dark). Line W145 shows the smallest difference between plants grown on a low-nitrate medium and plants grown on a high-nitrate medium. Line W705 shows the largest difference between the two growth media. The solid lines in Fig. 4 delineate low-NUpE and high-NUpE lines. In general, the between-line variability is greater for the low-NUpE lines than for the high-NUpE lines. (In Fig. 4 this corresponds to the top-left 7 × 7 square having a greater proportion of light squares than the bottom-right 10 × 10 square.) Interestingly, line W705 appears to be closer to the high-NUpE lines than to its fellow low-NUpE lines. The original field experiment to determine NUpE took place across two sites over two years. It is possible that a more detailed assessment involving data across more years would reclassify line W705 as a high-NUpE line.

In Fig. 4B, the lines are grouped by the level of nitrate in the medium as well as by NUpE. This enables a visual comparison of the effect of a change in nitrate level between the low- and high-NUpE lines. Comparing the top right-block of the upper-left 7 × 7 square with the top-right block of the bottom 10 × 10 square, we see that the effect of a change in nitrate level in the medium for the low-NUpE lines is larger than the effect for the high-NUpE lines. (The block corresponding to the former has a larger proportion of light squares.)

### Linear discriminant analysis (LDA) reveals which combinations of root traits determine NUpE

It is of interest to identify traits that discriminate between different groups of wheat roots. Approaches established in the literature for this problem include support vector machines (Iyer-Pascuzzi *et al.*, 2010) and logistic regression (Zurek *et al.*, 2015). The former involves finding hyperplanes that best separate the groups. In the method of Iyer-Pascuzzi *et al.* (2010), support vector machines are used to identify one or two traits that best distinguish between genotypes. Logistic regression can be used when there is a binary classification (B73 versus non-B73 maize founder populations in Zurek *et al.*, 2015.)

Given a data matrix with a number of explanatory variables and a response variable that is a grouping variable, linear discriminant analysis finds a linear combination of the explanatory variables that best discriminates between the groups. For the mathematical details of LDA see Supplementary Method S4.

**Fig. 5.**
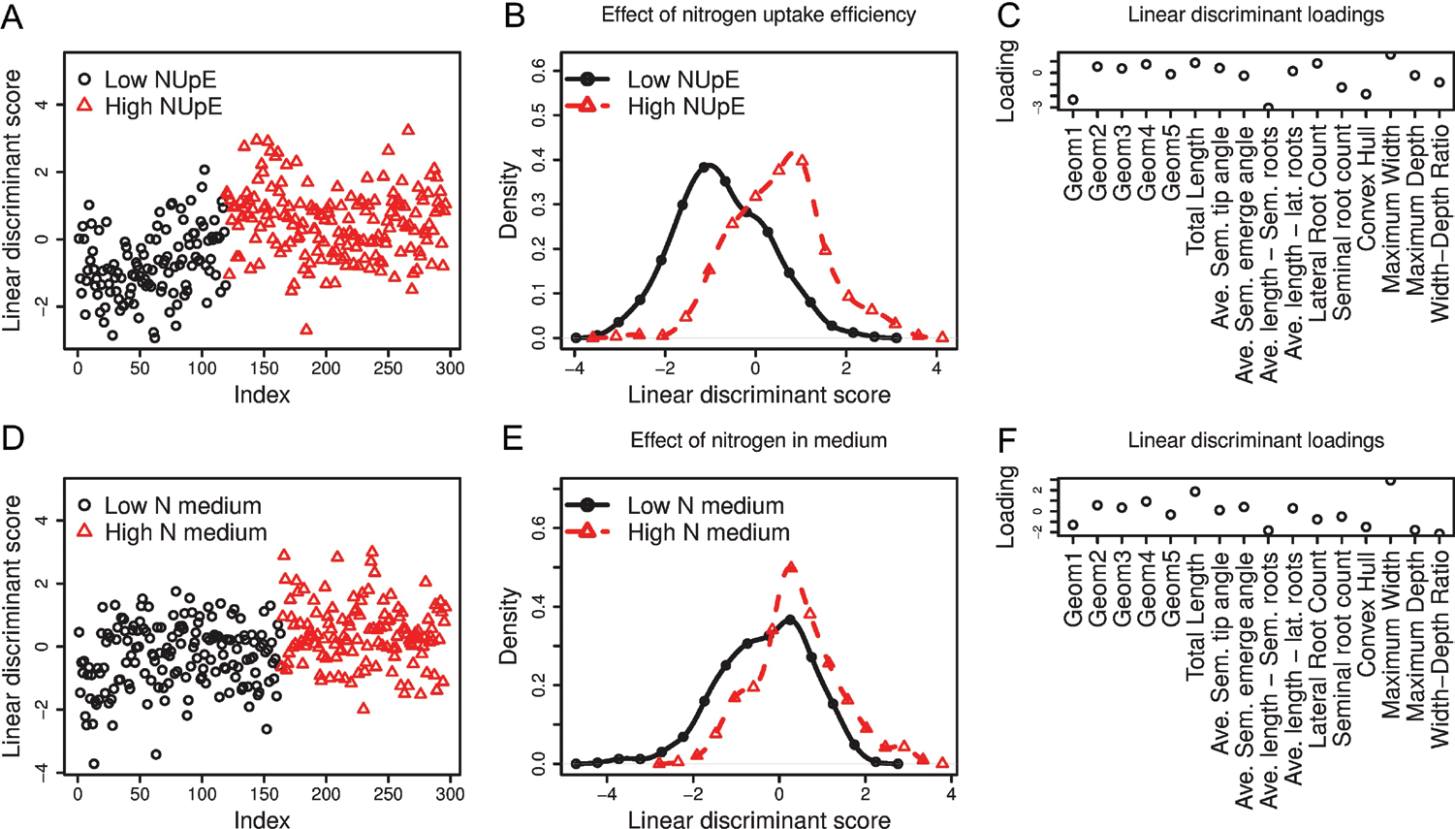
The use of linear discriminant analysis to separate wheat lines by nitrogen uptake efficiency (A–C) and by nitrate treatment (D–F). (A) The linear discriminant scores for low and high nitrogen uptake efficiency (NUpE) wheat lines. (B) Density plots of the linear discriminant scores in (A). (C) The loadings associated with each variable in the linear discriminant analysis comparing low- and high-NUpE lines. (D) The linear discriminant scores for low- and high-nitrate media. (E) Density plots of the linear discriminant scores in (D). (F) The loadings associated with each variable in the linear discriminant analysis comparing low- and high-nitrate media.

A comparison of individual traits with nitrogen uptake efficiency reveals no correlations (data not shown). The question of whether there are any differences in root system architecture (RSA) between the two rows (low versus high NUpE) or between the two columns (low versus high nitrate in medium) in Table 1 is now considered. The results of applying LDA to the data grouped in each of these two ways are shown in Fig. 5. It is clear from the density plots in Fig. 5B that LDA reveals significant differences between the RSA of low- and high NUpE-lines. The corresponding density plots for low versus high nitrate in the medium (Fig. 5E) show that although there is a modest difference between the two groups, it is not as clearly defined as for the NUpE.

**Table 1.** The codes and colours used in the figures for the data groups

	Low nitrate medium	High nitrate medium
Low NUpE	0 (black)	1 (red/magenta)
High NUpE	2 (green)	3 (blue)

The interpretation of Fig. 5C and 5F is as follows. The linear discriminant scores, the densities of which are plotted in Fig. 5B and 5E, are calculated by taking a weighted sum of the variables for each root system. Thus, in Fig. 5C the linear combination

–2.2Geom1+0.3Geom2+…–0.4Max Depth–1Width–Depth ratio

Density plots and the corresponding loadings vectors (i.e. vectors of weightings for the different variables) for the three linear discriminants that result from applying LDA to all four NUpE/nitrate treatment combinations in Table 1 are shown in Supplementary Fig. S3. The first linear discriminant (LD1), shown in Supplementary Fig. S3A, clearly discriminates between low-NUpE (black and red) and high-NUpE (green and blue) lines. The second linear discriminant (LD2), shown in Supplementary Fig. S3B, distinguishes between the low- and high-nitrate media for the low- and high-NUpE lines (black versus red). The third linear discriminant (LD3, Supplementary Fig. S3C) has little discriminatory power in this case.

The separation of the black and red lines in the density plot of LD2 corresponds to the earlier observation that the distances between low- and high-nitrate media samples for the low-NUpE lines are in general larger than for the high-NUpE lines (see Fig. 4B).

In order to explore which subsets of the variables could be used to explain the differences between the four NUpE/nitrate treatment combinations of Table 1, the subselect R package (https://cran.r-project.org/web/packages/subselect/index.html) was used. This is a package that addresses the issue of variable selection in different statistical contexts. The subselect R package allows for the rapid identification of the best subset of variables according to a particular index for subsets of size 1 to *p*–1, where *p* is the number of explanatory variables in the full model (16 in this case).

The score for the best subset of each size from 1 to *p*–1 is given in Supplementary Fig. S4. There are three features of this figure that deserve special mention. Firstly, the five MDS geometric variables (labelled Geom1–Geom5) all appear in the submodels for each submodel of size 9 or greater. This is evidence that there is useful information in these shape variables, and that the shapes of the root systems in the different groups are substantively different. Secondly, variable 10, average length of lateral roots, does not feature in any of the submodels. This is interesting since it suggests that it is not the lengths of the seedling lateral roots but the lateral root count that contributes to nitrogen uptake efficiency of the adult wheat root system. Thirdly, variable 11, lateral root count, features in every submodel. This highlights the importance of lateral roots in nutrient uptake.

**Fig. 6.**
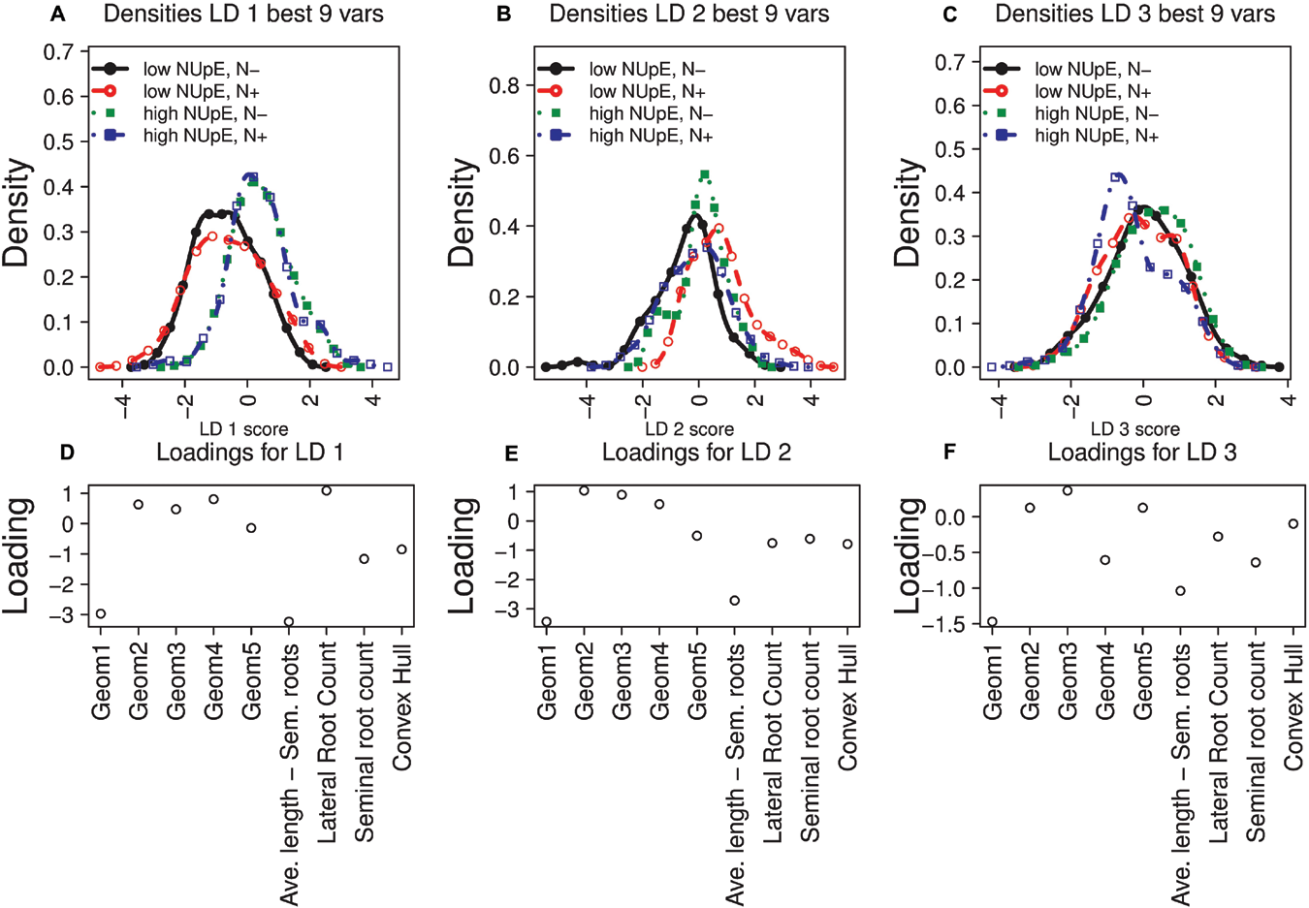
The densities and variable loadings for linear discriminant analysis using the best nine variables as determined by the ς^2^ (zeta2) coefficient from the subselect package in R. (A–C) Density plots of scores on linear discriminants (LD) 1–3 under the four NUpE/nitrate treatment conditions shown in Table 1. (D–F) Loadings vectors for LD1–3.

The results of performing LDA on the nine best variables obtained using the ς^2^ (zeta2) criterion of the subselect R package are shown in Fig. 6. These consist of the five geometric variables and the quantitative traits average length of seminal roots, lateral root count, seminal root count, and area of convex hull. Again, LD1 discriminates between low- and high-NUpE lines (Fig. 6A), and LD2 discriminates between low- and high-N media for low-NUpE lines (Fig. 6B). The third linear discriminant, LD3 (Fig. 6C), weakly distinguishes between low and high nitrate in the medium for high-NUpE lines (green and blue).

**Fig. 7.**
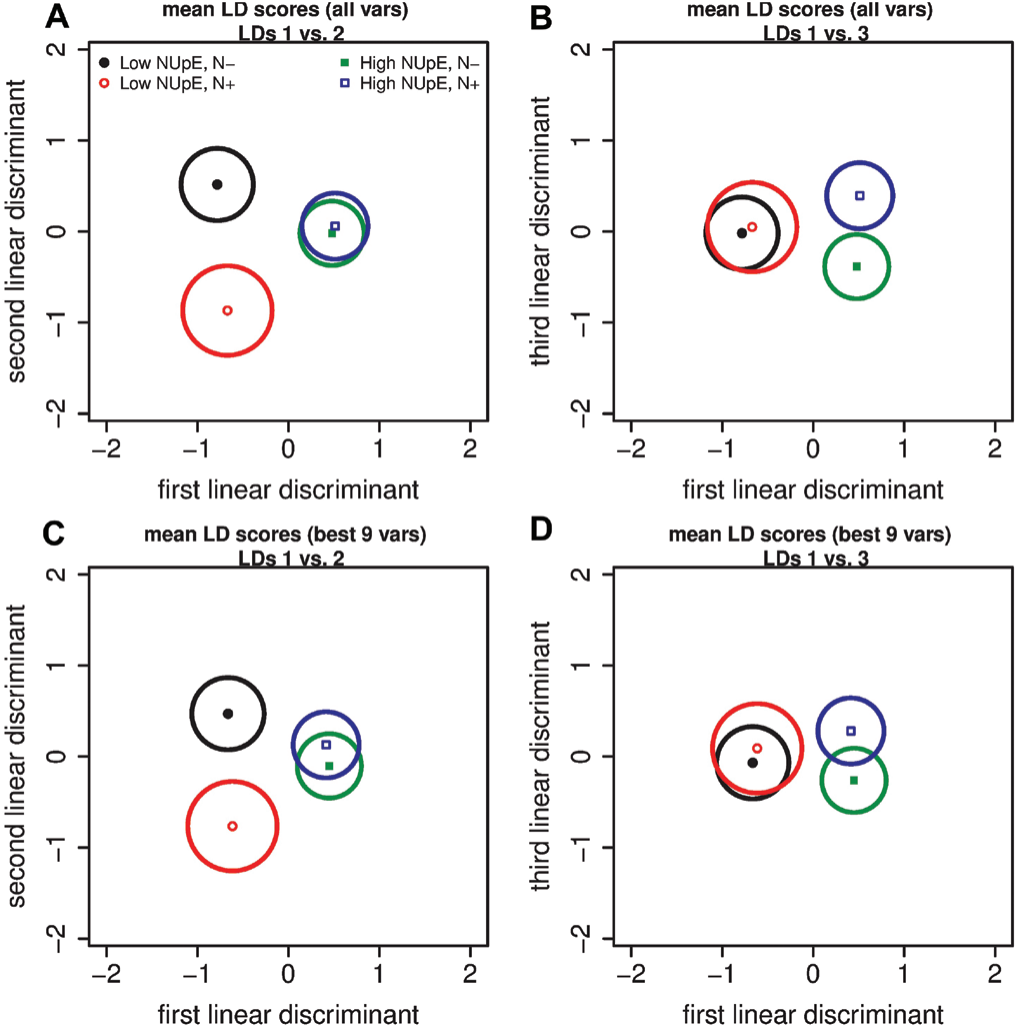
The mean linear discriminant scores with 99% confidence regions for the LDA with all variables (A, B) and with the subset of the best nine variables identified using the subselect package in R (C, D). (A) LD1 versus LD2 for all variables; (B) LD1 versus LD3 for all variables; (C) LD1 versus LD2 for the nine best variables; and (D) LD1 versus LD3 for the nine best variables

To present a clear, visual summary of the information in Supplementary Fig. S3 (LDA, all variables) and Fig. 5 (LDA, best nine variables), the mean linear discriminant (LD) scores for the four NUpE/nitrate treatment combinations with 99% confidence regions are given in Fig. 7. From Fig. 7A and 7B it is clear that the first LD using all 16 variables distinguishes between the low- and high-NUpE wheat plants, the second LD distinguishes between the low- versus high-nitrate treatment for low-NUpE plants, and the third LD distinguishes between the low- versus high-nitrate treatment for high-NUpE plants. All of these comparisons are significant at the 1% level. In Fig. 7C and 7D (best nine variables), the first two LDs serve the same role as when all of the variables are included, but the third LD fails to distinguish between the low- versus high-nitrate treatments for the high-NUpE plants (at the 1% level). It is to be expected that a LDA with fewer variables has a lower discriminatory power.

### Using bootstrapping to calculate the variability of the LDA coefficients (loadings)

For multivariate data, covariance matrices replace the variance term in a univariate analysis. In a bootstrap sample from a data matrix, high positive covariance between two variables indicates that when one variable is high the other variable is also high. Similarly, if the covariance is large in a negative direction, then there is an inverse relationship between the two variables.

Bootstrapping (see for example Good, 2005, for details) was used to explore the distribution and covariance structure of the loadings of the nine best variables. In bootstrapping, a data matrix is repeatedly generated from the original data matrix by taking a sample of size *N*, the number of plants in the original data set, with replacement, from the rows of the original data matrix. Sampling with replacement generates a data matrix with some repeated rows. For each sampled data matrix a LDA was carried out.

The distributions and covariance matrices (as heat maps) of the nine loadings with 1000 sampled data matrices are shown in Supplementary Figs S5 and S6, respectively. From Fig. S5 it is clear that the loadings on the variables Geom1 and average length of seminal roots are highly variable under resampling of the data matrix. In Fig. S6 we see that the loadings for Geom1 are strongly negatively correlated with the loadings for Geom2, Geom3, and Geom4, and strongly positively correlated with average length of seminal roots. In addition, the loadings of average length of seminal roots are highly positively correlated with the loadings for seminal root count.

Table 2 shows the mean loadings vectors with standard errors obtained by bootstrapping. For all three linear discriminants, all nine of the loadings are highly significantly different from zero (the *P*-value for area of convex hull on LD3 is 6.23 × 10^–7^, the *P*-value for Geom2 on LD3 is 1.23 × 10^–15^, and all other *P*-values are less than 1 × 10^–40^).

**Table 2.** Bootstrapped loadings vector means for linear discriminants 1, 2 and 3

Variable	LD 1	LD 2	LD 3
Geom1	–2.92 (0.037)	–2.94 (0.038)	–1.21 (0.042)
Geom2	0.64 (0.013)	0.87 (0.011)	0.14 (0.017)
Geom3	0.48 (0.009)	0.77 (0.008)	0.31 (0.011)
Geom4	0.78 (0.007)	0.48 (0.010)	–0.47 (0.009)
Geom5	–0.16 (0.006)	–0.42 (0.005)	0.11 (0.007)
Average length – seminal roots	–3.14 (0.034)	–2.30 (0.040)	–0.88 (0.044)
Lateral root count	0.99 (0.009)	–0.67 (0.013)	–0.21 (0.010)
Seminal root count	–1.12 (0.009)	–0.53 (0.014)	–0.52 (0.013)
Area of convex hull	–0.83 (0.010)	–0.67 (0.012)	–0.08 (0.016)

Standard errors in brackets. Means are based on the nine best variables for 1000 random samples with replacement of size *N*=296 of the rows of the original data matrix. The five variables Geom1–Geom5 are the output of the multidimensional scaling on the distance matrix of the root images. The *P*-values to test whether the loadings are significantly different from zero are all lower than 6.3 × 10^–7^.

### The use of permutation tests to assess the significance of the LDA of quantitative root traits

**Fig. 8.**
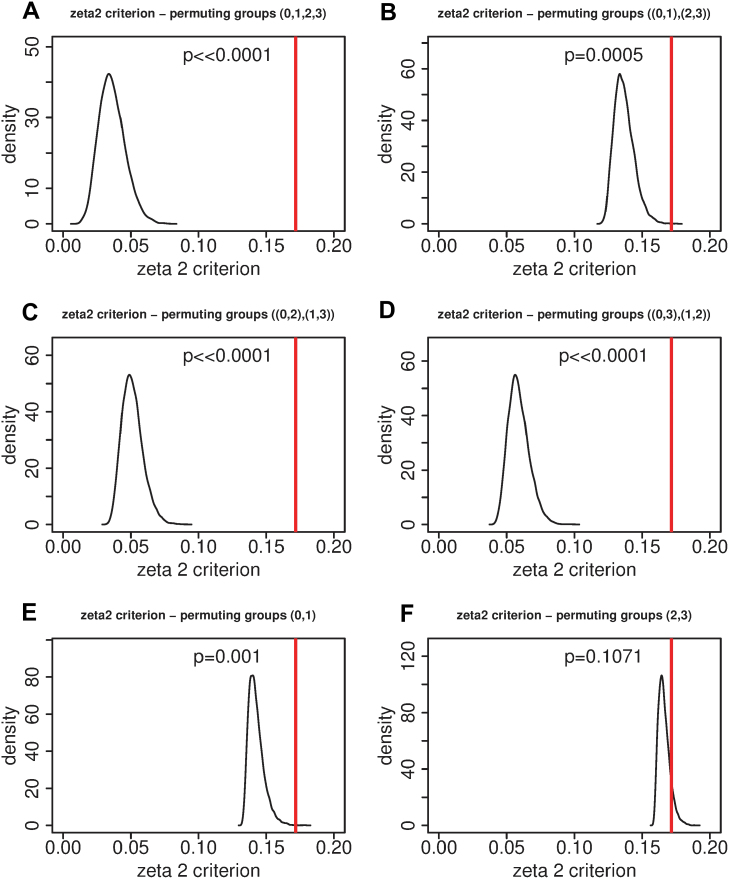
The ς^2^ (zeta2) criterion in permutation tests with *N*=10 000 permutations of the grouping variable. The vertical line indicates the result obtained with the true groupings. Group elements within brackets are permuted, so for example [(0,1),(2,3)] means that group labels are permuted within the subgroup (0,1) and within the subgroup (2,3). The meanings of the codes are: 0, low NUpE, low-nitrate medium; 1, low NUpE, high-nitrate medium; 2, high NUpE, low-nitrate medium; 3, high NUpE, high-nitrate medium. (A) Permuting group labels across all four groups. (B) Permuting group labels within low (0,1) and high (2,3) NUpE. (C) Permuting group labels within low-N (0,2) and high-N (1,3) media. (D) Permuting group labels within groups (0,3) and (1,2). (E) Permuting group labels within low NUpE, leaving high-NUpE group labels constant. (F) Permuting group labels within high NUpE, leaving low-NUpE group labels constant. The *P*-values show the probability of observing a zeta2 criterion as large as obtained with true group labels if the distribution obtained under permutation was the true distribution. (This figure is available in colour at *JXB* online.)

In order to assess the extent to which the LDA effectively distinguishes between the four NUpE/nitrate treatment combinations, a set of permutation tests was carried out (for a detailed statistical treatment of permutation tests see Good, (2005). In each test, the group labels across two or more of the groups were permuted 10 000 times and the LDA was re-run in each case. We used the ς^2^ (zeta2) condition for all of the tests. The results of permuting the grouping vector in six ways are shown in Fig. 8. The first four are (0,1,2,3), i.e. permuting all of the groups simultaneously (Fig. 8A); [(0,1),(2,3)], permuting within (0,1) and within (2,3), i.e. within the low- and high-NUpE plants, respectively (Fig. 8B) and, with the same notation, [(0,2),(1,3)] (Fig. 8C) and [(0,3),(1,2)] (Fig. 8D). Finally, for the last two permutations, first the group labels of (0,1) were permuted holding (2,3) constant (Fig. 8E), and then the group labels of (2,3) were permuted holding (0,1) constant (Fig. 8F).

The results of these permutation tests are striking, and Fig. 8A–D highlights the fact that the differences between low- and high-NUpE lines are much greater than the differences between low- and high-nitrate media. When permuting within either low- or high-NUpE, i.e. permutation [(0,1),(2,3)] (Fig. 8B), the results are much less significant than when we permute across the low/high NUpE boundary, permutations (0,1,2,3) (Fig. 8A), [(0,2),(1,3)] (Fig. 8C), and [(0,3),(1,2)] (Fig. 8D). The difference between low- and high-nitrate media for the low-NUpE lines, permutation (0,1) (Fig. 8E), is significant with *P*=0.001. Permutation (2,3) (Fig. 8F) shows that the difference between low- and high-nitrate media for the high-NUpE lines is not significant (*P*=0.1071).

### Visualizing the first linear discriminant to assess the effect of NUpE on root traits

**Fig. 9.**
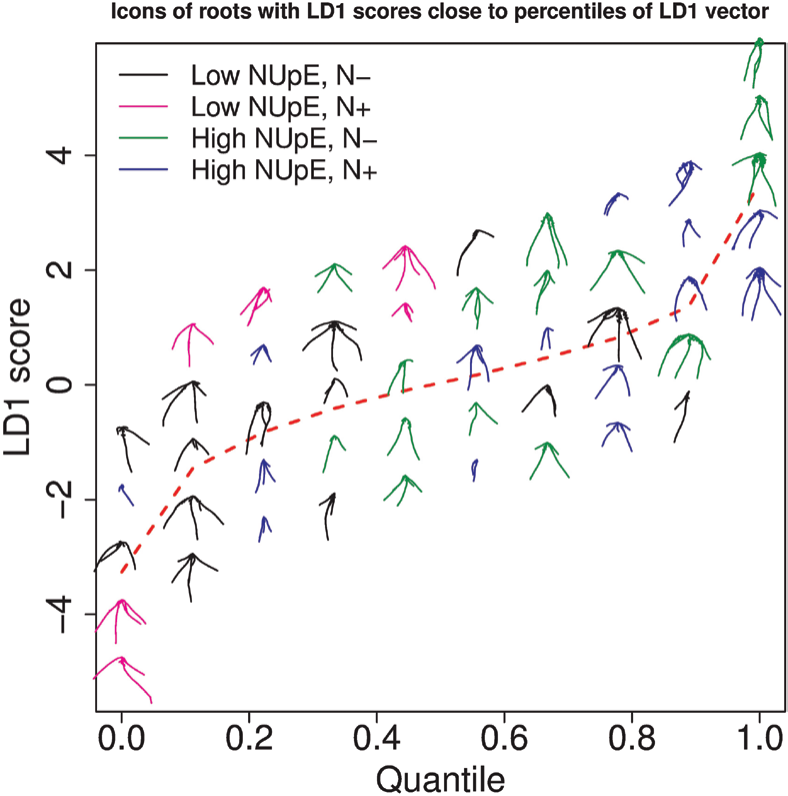
A visualization of the first linear discriminant (LD) on the nine best variables. Roots are plotted for which their LD1 score is close to the percentiles (0,0.1,0.2,…,1) of the LD1 vector. The dashed red line shows the percentiles of the LD1 vector. The vertical separation of the roots at a particular *x*-value is only for clarity of presentation.

To create Fig. 9, the percentiles (0,0.1,0.2,…,1) of the LD1 scores on the nine best variables were calculated. For each percentile, the five roots whose scores on LD1 were the closest to the percentile value were identified. Note that the vertical separation of the five roots above a particular *x*-value in Fig. 9 is only for clarity of presentation. The dashed red line joins the actual LD1 percentiles. The tendency of black and magenta plants (low NUpE) to have lower LD1 scores and green and blue plants (high NUpE) to have higher LD1 scores is again apparent from Fig. 9. Beyond this observation, it is difficult to discern any particular pattern in the shapes of the roots by eye as we move from left to right in the figure. For an elaboration of the reasons behind the heterogeneity in root shape at a particular *x*-value in the figure, see the Discussion.

The reason for including this figure is to highlight that although LDA clearly distinguishes between low- and high-NUpE lines (Figs 5, 6, and 7), the differences picked up by this statistical analysis are not readily discriminated by eye. This poses a challenge to the identification of a particular ideotype for improved nitrogen uptake efficiency and suggests, unsurprisingly, that one cannot look at root system architecture in isolation in order to establish such an ideotype.

## Discussion

The question of how the root system architecture (RSA) of wheat plants differs between high and low nitrogen uptake efficiency (NUpE) lines and between different nitrate levels in the growth medium has been addressed. Only the shape features on the root systems were considered, and the data set consisted of 2-D measurements on approximately 300 wheat plants. Linear discriminant analysis (LDA) was used to construct composite variables (linear combinations of the original variables) that discriminate effectively between the different groups of wheat seedlings (low versus high NUpE and low versus high nitrate in medium). This demonstrated structural differences in the RSA of wheat lines by NUpE.

It is important to recognize that differences between plants with different nutrient uptake efficiencies may not be in macroscopic morphological traits such as root lengths, numbers of lateral roots, or area of convex hull. Specifically, there may be anatomical or physiological differences that were not considered in this study, for example the number or size of aerenchyma spaces within the seminal roots. Equally, there may be differences in the number or distribution of root hairs, another feature that has not been taken into consideration. There may also be metabolic, nutrient uptake or storage differences between lines. This analysis used wheat seedlings; there may be root traits important for NUpE that only appear at later growth stages. If a study were to be conducted that included an analysis of traits such as aerenchyma spaces or root hairs in addition to the variables used in this analysis, it would be straightforward to include these variables in the data matrices for the linear discriminant analysis, by including additional columns for each of the new variables.

Linear discriminant analysis revealed clear differences in the distributions of the linear discriminant scores between low- and high-NUpE wheat lines (Figs 5, 6, and 7). Linear combinations of the quantitative traits included in the data set that discriminate between the groups were identified. These linear discriminants are functions of all of the variables, making it difficult to deduce easily identifiable traits. In order to understand the output of LDA, and to explain the heterogeneity of the observed root shapes for a given score on the relevant linear discriminant in Fig. 9, consider a two-variable system. Suppose two traits, X_1_ and X_2_, are measured, and LDA reveals that 3X_1_ – X_2_ is the linear combination of the variables that best distinguishes between the groups. Consider the set of possible (X_1_,X_2_)-coordinates for which 3X_1_ – X_2_ = 0. In this two-variable context, the set of solutions is the line X_2_ = 3X_1_. With more variables, the solution set for a particular value of the linear discriminant is a plane (for three variables) or a hyperplane (for four or more variables). It is therefore not surprising that there is no clear phenotypic pattern in RSA if the roots are ordered by their score on a particular linear discriminant, as in Fig. 9.

With this explanation in mind, the question of how useful LDA is as a tool for identifying desirable traits in plant phenotyping analysis is now considered. The differences in the distributions of the linear discriminant scores in Figs 3 and 4 are striking and highly statistically significant (see Fig. 8 for the results of permutation tests to assess the level of significance). Clearly there are structural differences in the data corresponding to low- and high-NUpE lines.

The shape variables, obtained from the multidimensional scaling, make an important contribution to the discriminatory power of the LDA. This is shown by the fact that the best subsets of variables of sizes 9 and above all contain all five of the MDS variables (see Supplementary Fig. S4 and the Results section). This shows that the shapes of the root systems in different NUpE/nitrate treatment combinations are genuinely different. In addition, again from Supplementary Fig. S4, the variable corresponding to average length of lateral roots does not feature in any of the submodels. On the contrary, lateral root count is an element of all the best submodels, irrespective of number of variables. So, in this analysis, lateral root density emerges as a more significant contributor to a wheat root’s NUpE than the number of lateral roots.

Localized N supply has been shown to promote first- and second-order root branching in numerous crop species (Drew, 1975). More recently, root branching responses to N availability in wheat have been shown to be genotype-dependent. Melino *et al.* (2015) tested a panel of various wheat genotypes under low N and found that although all genotypes increased root surface area in response, some did this by increasing lateral root count while others increased total root length. In maize (*Zea mays*), it has been shown that fewer, long lateral roots is optimal for N acquisition in suboptimal N conditions (Zhan and Lynch, 2015). Mean primary root length, lateral root count, seminal root count, and convex hull were all included in the three models best able to discriminate low- and high-NUpE genotypes. However, mean lateral root length was not. This may be due to the mean value being a rather simplistic metric for lateral root function that does not take into account lateral root density. A useful output of shape analysis studies is an increased understanding of which traits (or trait combinations) are of most importance in linking root architecture to function, information which can then be used to inform the design of improved phenotyping pipelines. In this case, future image analysis tools will be designed to measure lateral root density profiles as well as number and length.

Phenotyping crop root systems under field conditions is technically challenging and most high-throughput screens thus utilize controlled-environment conditions and pot-grown plants or seedlings (e.g. Bai *et al.*, 2013; Clark *et al.*, 2013; Atkinson *et al.*, 2015). Seedling screens offer the highest throughput and are more amenable to automated quantification but are of limited benefit unless seedlings traits impact adult plant performance. Seedling root traits have been found to correlate with field performance in maize (Li *et al.*, 2015), spring wheat (Watt *et al.*, 2013), and durum wheat (Canè *et al.*, 2014). A key finding of the work presented here is that combinations of seedling root traits not readily observable by eye can be related to the field performance of mature plants.

In theory, all of the information about the RSA is contained in the co-ordinates of the smooth curves fitted to the seminal and lateral roots. Could this information alone be used to elicit more practical information about the features of the low- and high-NUpE roots that confer the differences that our analysis has revealed? One approach that may work is to make use of the ideas in Feragen *et al.* (2011). This approach to working with mathematical trees, of which root systems are an example, is to use topological considerations. In this current work, a metric developed in a so-called quotient space, in which trees that share the same topology are collapsed to a single point, would allow for a universal co-ordinate system for the roots in a data set. With this in place, it would be possible to use PCA directly on the shapes of the roots (rather than on the derived traits as we did in our analysis). This may lead to the elicitation of more readily discernible differences in RSA between low- and high-NUpE wheat lines. Computationally, the approach using a quotient metric is demanding and there is no readily available computer software to make the necessary calculations. But it is certainly an area worth exploring, and it will guide future work. Principal components, like linear discriminants, are formed by constructing linear combinations of the original variables. However, unlike for LDA, which works on grouped data, PCA can be applied to ungrouped data. It would be interesting to create a universal (or global) system of co-ordinates of wheat roots based on the quotient tree metric of Feragen *et al.* (2011) and observe whether high- versus Low-NUpE plants cluster together in principal component space. Without carrying out the (computationally demanding) work, it is not possible to say whether principal components used in this way would offer an approach to the selection of N-efficient lines. However, an advantage of this approach would be that it would be possible to construct sets of images of roots obtained by travelling along particular principal components, and it may be easier to establish qualitatively what each principal component corresponds to in terms of root morphology than to assess what linear discriminants correspond to.

In conclusion, one key finding of this paper is that the distribution of seedling RSA traits between wheat plants classified as high- and low-NUpE in field trials are highly significantly different. To a lesser extent, for low-NUpE plants, there is a difference in distributions of RSA traits for seedlings grown on low- versus high-nitrate media. In this case linear discriminant analysis was used to elucidate these differences. The variables that emerged as significant in this analysis were average length of seminal roots, lateral root density, seminal root count, and convex hull area, as well as geometric variables that capture the morphology of the roots. Some possible mechanisms by which the traits identified can explain the differences in NUpE of plants are offered. Linking combinations of seedling root traits to mature plant field performance using the techniques presented here is a potential solution to the challenge of high-throughput functional phenotyping of plant root systems.

## Supplementary Data

Supplementary data are available at *JXB* online.

Table S1. The quantitative variables (unscaled, raw data).

Table S2. The quantitative variables (scaled to have mean 0 and variance 1).

Fig. S1. The NUpE data for the two growing seasons 2010–11 and 2011–12.

Fig. S2. The first three principal components of the data matrix.

Fig. S3. Density plots and loadings vectors for the three linear discriminants using the four groups in Table 1.

Fig. S4. A plot showing the best LDA sub model using 1,2,…,*p*–1 variables.

Fig. S5. Bootstrapped distributions of the loadings of the nine best variables for linear discriminants 1–3.

Fig. S6. The correlation matrices for the loadings on the nine best variables under bootstrapping as in Fig. S5.

Method S1. Distance measure between root systems.

Method S2. Multidimensional scaling.

Method S3. Mahalanobis distance.

Method S4. Linear discriminant analysis.

## Supplementary Material

Supplementary Material
