# Supplementary material for: Linear discriminant analysis reveals differences in root architecture in wheat seedlings related to nitrogen uptake efficiency
Source: J Exp Bot. 2017 Sep 27;68(17):4969–81. doi: 10.1093/jxb/erx300 (PMC5853436; doi:10.1093/jxb/erx300)
Supplement: Supplementary Material [file erx300_suppl_supplementary_meterial.zip › Supplementary_Methods_S1_S4_Figures_S1_S6.pdf]

Supplementary Material for "Linear discriminant analysis reveals differences in root system architecture in wheat seedlings related to nitrogen uptake efficiency"

Kenobi K[1], Atkinson J[2], Wells DM[2], Gaju O[3], deSilva JG[3], Foulkes J[3], Dryden IL[2,4], Wood ATA[2,4] and Bennett MJ[2,3]

[1]Department of Mathematics, Aberystwyth University, Penglais, Aberystwyth, Ceredigion, SY23 3BZ
[2]Centre for Plant Integrative Biology, Sutton Bonington Campus, University of Nottingham, LE12 5RD, UK
[3]Division of Plant and Crop Sciences, Sutton Bonington Campus, University of Nottingham, LE12 5RD, UK
[4]School of Mathematical Sciences, University Park, University of Nottingham, NG7 2RD, UK

Supplementary Method S1: Distance measure between root systems

In this section a distance measure between two root systems is described. The first step is to identify a set of n equally spaced landmarks along each seminal root and a set of k equally spaced landmarks along each lateral root. Then, given two root systems, in the full distance measure optimally match the seminal roots and the lateral roots. However, this full distance measure is very computationally intensive. So, since information about number and length of lateral roots is also included in the quantitative data available from the RootNav analysis, a partial distance measure that only takes into consideration the shapes of the seminal roots is also presented.

Comparing individual seminal roots

Let X_1 and X_2 denote two seminal roots with lateral roots (LRs). Let

$$X_i = (r_i, s_{Li}, x_{Li}, s_{Ri}, x_{Ri}, l_i), \text{ for } i = 1, 2.$$

Here, $r_i \in (\mathbb{R}^2)^{N-1}$ is an $(N-1) \times 2$ matrix of the coordinates of N equally spaced landmarks along the ith seminal root after subtraction of the first landmark. The vectors $s_{\kappa i} \in [0,1]^{N_{\kappa i}}$ for $\kappa \in \{L, R\}$ are the positions of the start points of the $N_{\kappa i}$ LRs on the left ($\kappa = L$) and right ($\kappa = R$) of the seminal root as a proportion of the seminal root length, l_i. The arrays $x_{\kappa i}$ are of dimension $(K-1) \times 2 \times N_{\kappa i}$, and the jth slice of $x_{\kappa i}$ contains the coordinates of K landmarks along the jth LR again after subtraction of the first landmark.

An optimal one-to-one mapping between the LRs of the two seminal roots is found as follows. For ease of notation, let $\sigma_\kappa \in \{1, 2\}$ represent the index of the seminal root with fewest LRs on side $\kappa \in \{L, R\}$ and τ_κ to be the other element of $\{1, 2\}$. Note that if $N_{\kappa 1} = N_{\kappa 2}$ we set $\sigma_\kappa = 1$. Then $N_{\kappa\sigma_\kappa} \leq N_{\kappa\tau_\kappa}$ for $\kappa \in \{L, R\}$.

Let

$$f_\kappa : \{1, 2, \ldots, N_{\kappa\sigma_\kappa}\} \mapsto A(f_\kappa) \subseteq \{1, 2, \ldots, N_{\kappa\tau_\kappa}\},$$

where $f_\kappa(1) < f_\kappa(2) < \ldots < f(N_{\kappa\sigma_\kappa})$, be a one-to-one, order-preserving mapping from the index set of the LRs on side κ of X_{σ_κ} to a subset of the index set of the LRs on side κ of X_{τ_κ}.

Given a pair of mappings, (f_L, f_R), what is the cost of matching the LRs on the left of X_{σ_L} to the LRs on the left of X_{τ_L} indexed by $A(f_L)$ and the LRs on the right of X_{σ_R} to the LRs on the right of X_{τ_R} indexed by $A(f_R)$? A penalty based on three quantities is imposed:

1. The sizes of the differences in the positions of the matched LRs (calculated using the elements of the s vectors and the lengths of the seminal roots)

2. The Euclidean distances between the matched LRs

3. The Euclidean norms of the unmatched LRs.

The differences between the shapes of the seminal roots themselves also need to be accounted for. Define

$$d^2_{f_L,f_R}(X_1, X_2) = \|r_1 - r_2\|^2 + \sum_{\kappa \in \{L,R\}} \left(\sum_{j=1}^{N_{\kappa\sigma_\kappa}} \left(|s_{\kappa\sigma_\kappa j} l_{\sigma_\kappa} - s_{\kappa\tau_\kappa f_\kappa(j)} l_{\tau_\kappa}|^2 + \|x_{\kappa\sigma_\kappa j} - x_{\kappa\tau_\kappa f_\kappa(j)}\|^2 \right) + \sum_{j \in B(f_\kappa)} \|x_{\kappa\tau_\kappa j}\|^2 \right), \quad (1)$$

where $B(f_\kappa) = \{1, 2, \ldots, N_{\kappa\tau_\kappa}\} \setminus A(f_\kappa)$ for $\kappa \in \{L, R\}$ is the index set of the unmatched LRs in X_{τ_κ}.

Minimising over all possible mappings, f_L and f_R, yields

$$d^2(X_1, X_2) = \min_{f_L, f_R} d^2_{f_L,f_R}(X_1, X_2). \quad (2)$$

The mappings at which the minimum distance is obtained are denoted $\hat{f}_L$ and $\hat{f}_R$.

The distance measure, $d(\cdot,\cdot)$, is the full distance measure (including lateral roots) between two seminal roots. For a computationally less demanding distance measure, restrict attention to the seminal roots. For two seminals, X_1 and X_2,

$$\tilde{d}^2(X_1, X_2) = \|r_1 - r_2\|^2. \tag{3}$$

Comparing two root systems with multiple seminal roots

Let $Y_i = (Y_{i1}, Y_{i2}, \ldots, Y_{iM_i})$ be an ordered list of M_i seminal roots with LRs for $i = 1, 2$. Let $\sigma \in \{1, 2\}$ be the index of the root system with fewest seminal roots and $\tau \in \{1, 2\}$ to be the other index. If $M_1 = M_2$ set $\sigma = 1$.

Let $g : \{1, 2, \ldots, M_\sigma\} \mapsto A^{'}(g) \subseteq \{1, 2, \ldots, M_\tau\}$, with $g(1) < g(2) < \ldots < g(M_\sigma)$, be an order-preserving, one-to-one mapping between the index set of the seminals in Y_σ to a subset of the index set of the seminals in Y_τ.

The root Y_σ is 'zero-padded' by inserting $M_\tau - M_\sigma$ 'null seminals' such that these null seminals are indexed by $B^{'}(g) = \{1, 2, \ldots, M_\tau\} \setminus A^{'}(g)$ and the non-null seminals are indexed by $A^{'}(g)$. In order not to penalise differences in the position of LRs when we compare these null seminals with the unmatched seminals in Y_τ, the elements s_L, s_R and l in the null seminals are matched with the corresponding elements of their counterparts, i.e. the unmatched seminals, in Y_τ.

Given a mapping g, define a distance

$$d_g^2(Y_1, Y_2) = \sum_{j=1}^{M_\sigma} d^2(Y_{\sigma j}, Y_{\tau g(j)}) + \sum_{j \in B^{'}(g)} \|Y_{\tau j}\|_{s=}^2,$$

where

$$\|(r, s_L, x_L, s_R, x_R, l)\|_{s=}^2 = d^2((r, s_L, x_L, s_R, x_R, l), (0, s_L, 0, s_R, 0, l)),$$

and $d^2(\cdot,\cdot)$ is defined in (2). Define

$$\begin{aligned} d_{TOT}^2(Y_1, Y_2) &= \min_g d_g^2(Y_1, Y_2), \qquad \text{and let} \\ \hat{g}(Y_1, Y_2) &= \operatorname*{argmin}_g d_g^2(Y_1, Y_2) \end{aligned}$$

be the optimal matching function between the seminal roots of Y_1 and Y_2.

For the seminal only measure, define

$$\tilde{d}_{TOT}^2(Y_1, Y_2) = \min_g \tilde{d}^2(Y_1, Y_2), \tag{4}$$

where

$$\tilde{d}_g(Y_1, Y_2) = \sum_{j=1}^{M_\sigma} \tilde{d}^2(Y_{\sigma_j}, Y_{\tau g(j)}) + \sum_{j \in B^{'}(g)} \|r(Y_{\tau j})\|^2, \tag{5}$$

and $r(Y_{\tau j})$ is the $(n-1) \times 2$ matrix of coordinate points defining the seminal root of $Y_{\tau j}$.

Supplementary Method S2: Multidimensional scaling

For the N wheat plants in the data set, the measurements $x_{1i}, x_{2i}, \ldots, x_{pi}$ for $i =, \ldots, N$ on the p quantitative variables generated by the RootNav software (Pound et al., 2013) are available. In addition d geometric variables, $X_1, \ldots, X_d$, are constructed by applying multidimensional scaling (MDS) to the distance matrix, $\Delta = (\delta_{ij})$, where

$$\delta_{ij} = \sqrt{d^2_{TOT}(Y_i, Y_j)},$$

the distance between the ith and jth roots as measured by d_{TOT}, which is defined above. Given a distance matrix, MDS creates a set of points in a d-dimensional Euclidean space for a pre-specified number of dimensions, d, for which the Euclidean distances between these points are as close as possible to the inter-point distances given in the distance matrix. For details of the MDS algorithm see for example Mardia, Kent and Bibby (1979), Chapter 14, pp. 396-493.

Supplementary Method S3: Mahalanobis Distance

The Mahalanobis distance between two populations with means μ_1 and μ_2, and common covariance matrix $\mathbf{\Sigma}$ is given by Δ, where

$$\Delta^2 = (\mu_1 - \mu_2)^T \mathbf{\Sigma}^{-1} (\mu_1 - \mu_2).$$

The corresponding sample Mahalanobis distance, D, is given by

$$D^2 = (\bar{\mathbf{x}}_1 - \bar{\mathbf{x}}_2)^T \mathbf{S}^{-1} (\bar{\mathbf{x}}_1 - \bar{\mathbf{x}}_2),$$

where $\bar{\mathbf{x}}_i$ for $i = 1, 2$ is the sample mean and $S = (n_1 \mathbf{S}_1 + n_2 \mathbf{S}_2)/(n-2)$ is an unbiased estimator for the common covariance matrix, $\mathbf{\Sigma}$.

For further details see Mardia, Kent and Bibby (1979), pp. 76-79.

Supplementary Method S4: Linear Discriminant Analysis

The data are in four groups, coded 0, 1, 2 and 3. These correspond to the two factors nitrogen uptake efficiency (NUpE) and nitrogen in medium. The codes identify the groups as shown in Table 1 of the main text. Linear discriminant analysis (LDA) finds linear combinations of the explanatory variables that best discriminate between the different groups. The description given on pages 331-332 of Venables and Ripley (2002) is presented. Let W

denote the within-group covariance matrix, that is the covariance matrix of the variables centred on the group mean, and B denote the between-group covariance matrix, that is, of the predictions by the group means. Let M be the $g \times p$ matrix of group means, and G be the $n \times g$ matrix of group indicator variables (so $g_{ij} = 1$ if and only if case i is assigned to group j). Then the predictions are GM. Let $\bar{\mathbf{x}}$ be the means of the variables over the whole sample. Then the sample covariance matrices are $W = ((X - GM)^T(X - GM))/(n - g)$ and $B = ((GM - \mathbf{1}\bar{\mathbf{x}})^T(GM - \mathbf{1}\bar{\mathbf{x}}))/(g - 1)$, where $\mathbf{1}$ is a vector of ones. Then B has rank at most $\min(p, g - 1)$. Fisher's two-class discriminant analysis involves finding linear combinations of the variables, $\mathbf{a}^T x$ for which the ratio $\mathbf{a}^T B\mathbf{a}/\mathbf{a}^T W\mathbf{a}$ is maximized (see for example Mardia et al., 1979, pp. 318-319 for details). LDA is an extension of the two class case and involves finding eigenvalues and corresponding eigenvectors of the asymmetric matrix $W^{-1}B$.

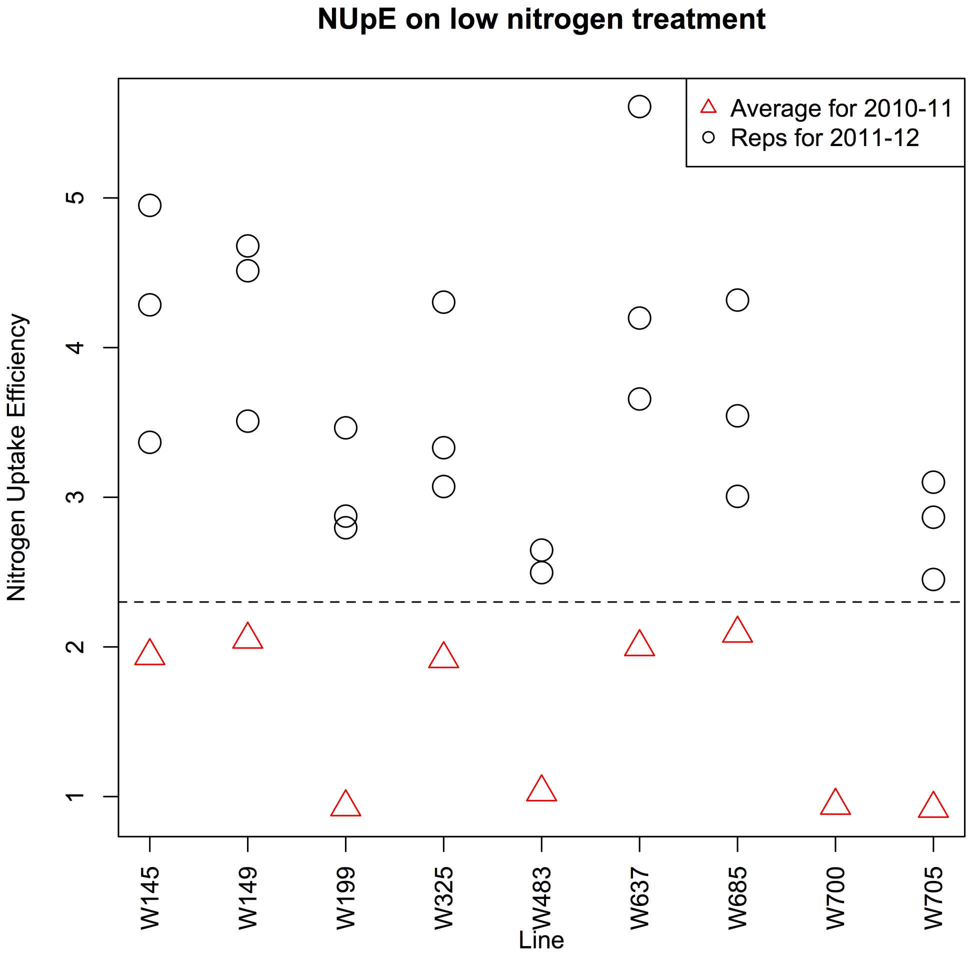

Figure S1: The NUpE data for the two growing seasons 2010-11 and 2011-12. The nine lines were selected on the basis of their NUpE on a low nitrogen treatment in season 2010-11. On this basis, lines W199, W483, W700 and W705 were classified as low NUpE and lines W145, W149, W325, W637 and W685 were classified as high NUpE. The data from 2011-12 were used to confirm that the patterns observed were robust across different years. Note that in 2011-12 no data are available for line W700 and W483 has only two replicates.

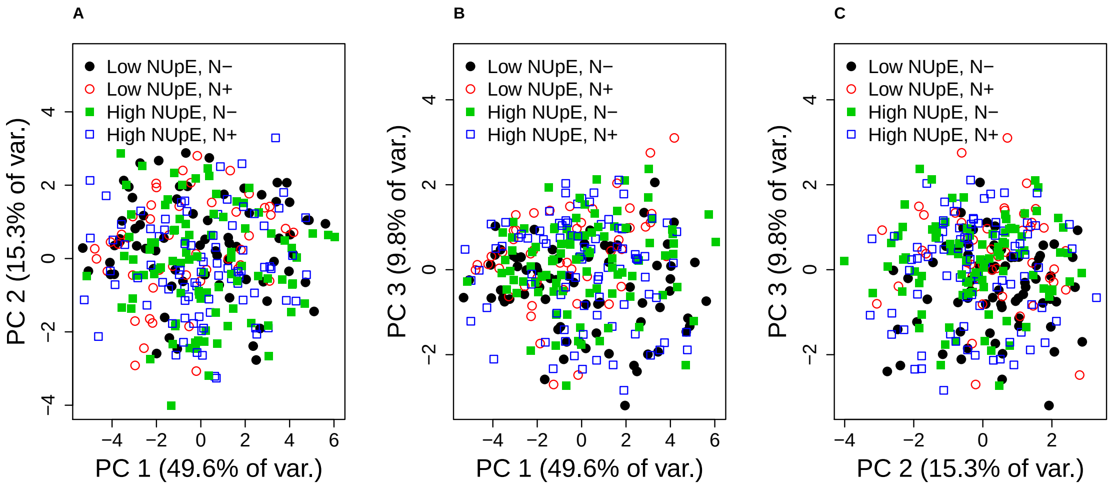

Figure S2: The first three principal components of the data matrix. The three plots show respectively the scores on A) PC1 against PC2, B) PC1 against PC3 and C) PC2 against PC3

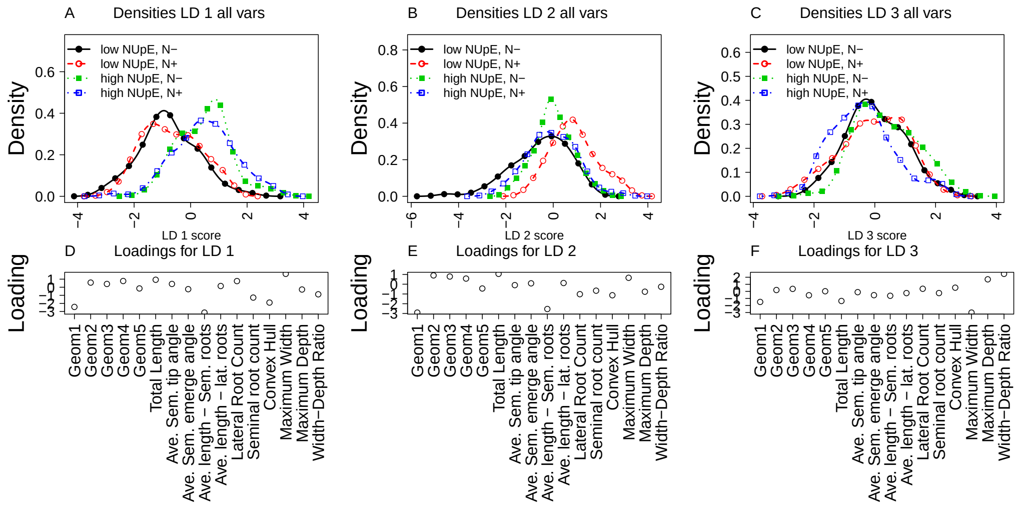

Figure S3: Density plots (A-C) and loadings vectors (D-F) for the three linear discriminants using the four groups in Table 1 of the main text.

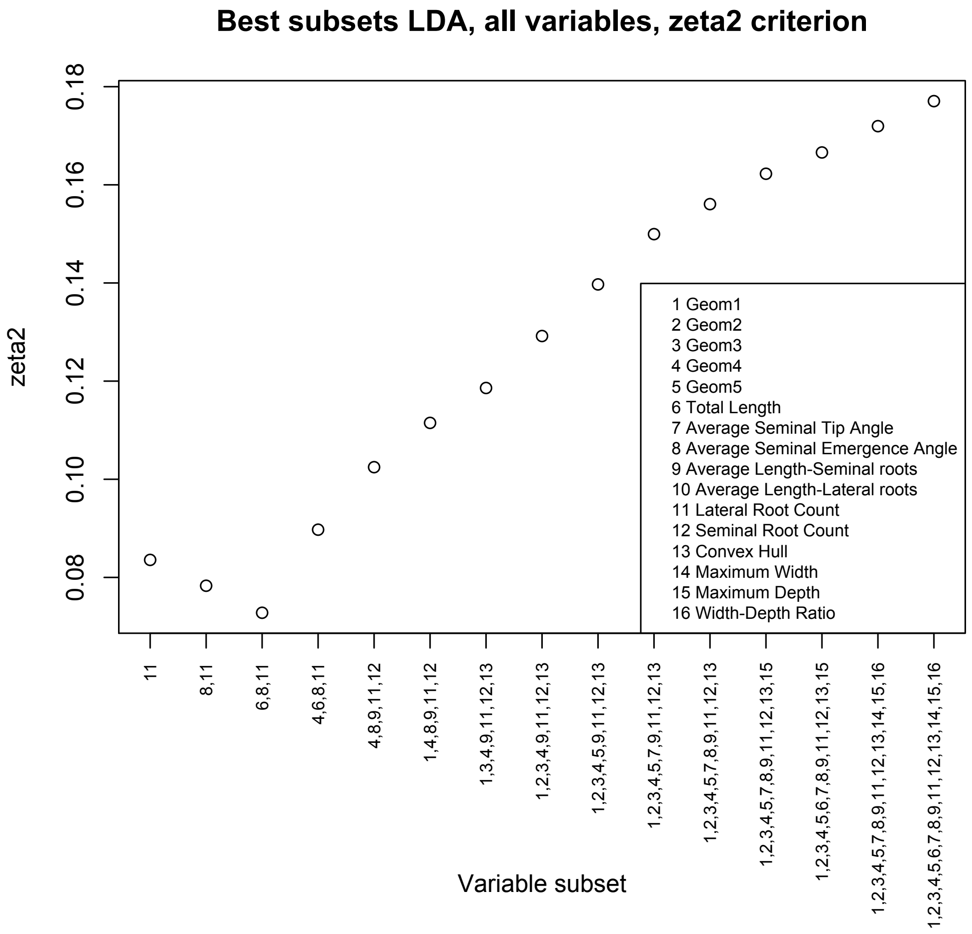

Figure S4: A plot showing the best LDA sub model using $1, 2, \ldots, p-1$ variables. Note that variable 11, lateral root count features in every sub model, all five of the geometric variables appear in every sub model of size 9 or larger and variable 10, average length of lateral roots does not feature in any of the sub models

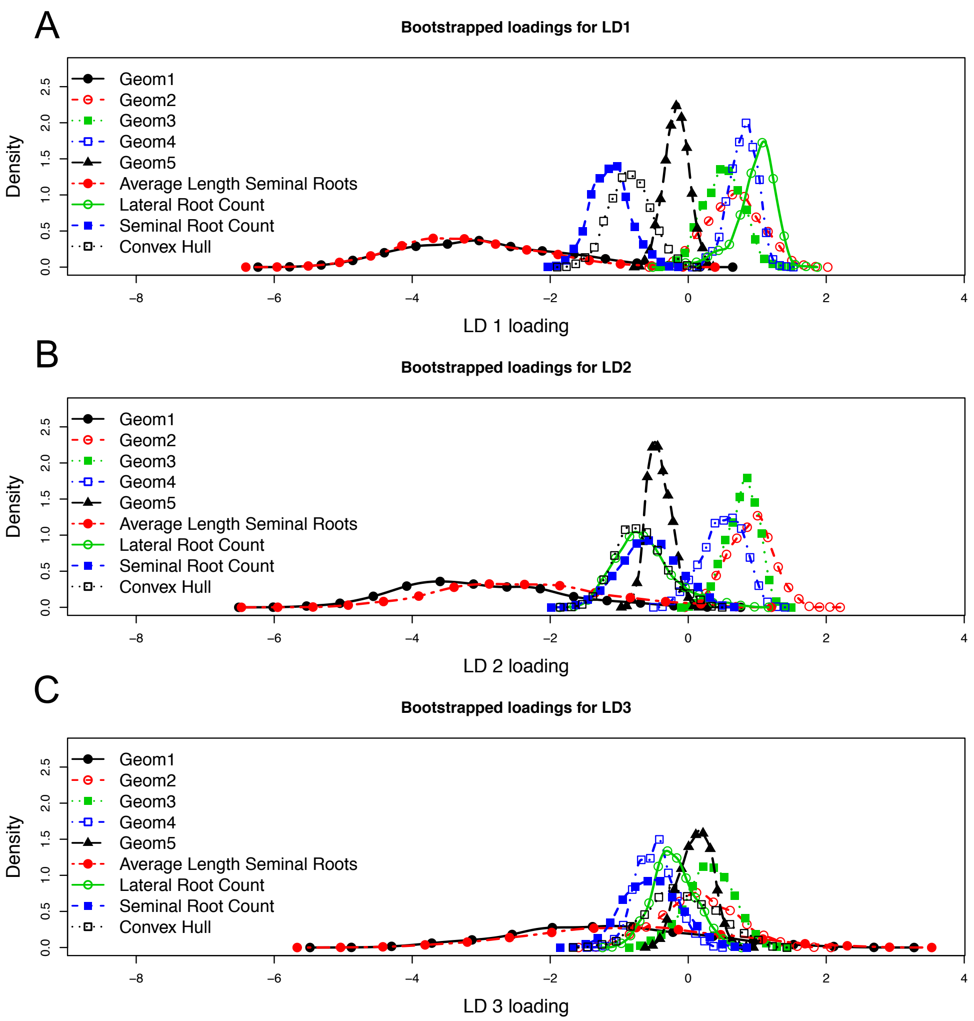

Figure S5: Bootstrapped distributions of the loadings of the nine best variables for linear discriminants 1 (A), 2 (B) and 3 (C). A sample of N=296 rows of the data matrix was generated 1000 times with replacement and the loadings for the three linear discriminants were calculated for each sample

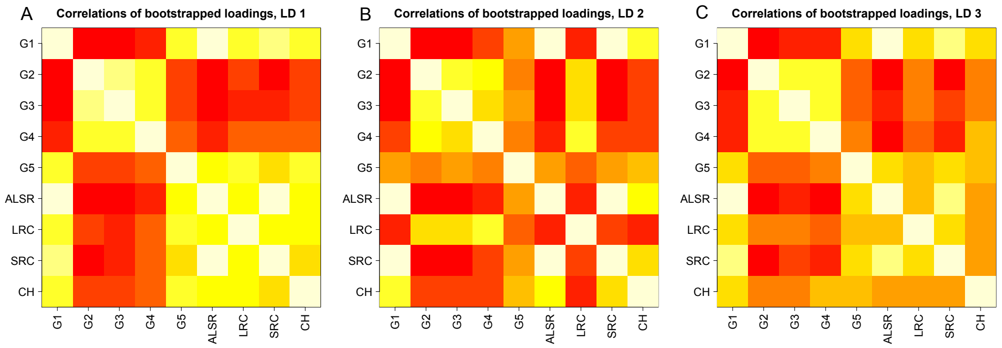

Figure S6: The correlation matrices for the loadings on the nine best variables under bootstrapping as in Figure S5. A) Linear discriminant 1 B) Linear discriminant 2 C) Linear discriminant 3. Here the dark red squares correspond to strong negative correlation and the light squares to strong positive correlation. The abbreviations are as follows: G1-G5: Geom 1 to Geom 5, ALSR: Average length - seminal root, LRC: Lateral root count, SRC: Seminal root count, CH: Convex hull
